# A Dashboard for Managing an Ecosystem and People With Dementia: Protocol for a Healthy Ageing Ecosystem for People With Dementia (HAAL) International Feasibility Pilot Study

**DOI:** 10.2196/59860

**Published:** 2025-08-06

**Authors:** Giulio Amabili, Elvira Maranesi, Federico Barbarossa, Arianna Margaritini, Anna Rita Bonfigli, Fong-Chin Su, Chien-Ju Lin, Hsiao-Feng Chieh, Dianne Vasseur, Henk Herman Nap, Yeh-Liang Hsu, Dorothy Bai, Roberta Bevilacqua

**Affiliations:** 1 Scientific Direction IRCCS INRCA Ancona Italy; 2 Medical Device Innovation Center (MDIC) National Cheng Kung University Tainan City Taiwan; 3 Vilans—National Expertise Centre on Long-Term Care Utrecht The Netherlands; 4 School of Gerontology and Long-Term Care, College of Nursing Taipei Medical University Taipei Taiwan

**Keywords:** older people, dementia, dashboard, technology for older adults, digital health, eHealth, innovation in health care

## Abstract

**Background:**

Dementia is a syndrome characterized by a wide spectrum of symptoms and needs. There is no cure for this syndrome, which represents a major challenge to society in terms of quality of life for those affected and in terms of workload and stress burden for those who take care of them.

**Objective:**

The Healthy Ageing Ecosystem for People With Dementia (HAAL) aimed to improve the quality of life of both people with dementia and their formal caregivers (FCs) and informal caregivers (ICs) by providing a personalized set of devices to the person with dementia, along with a dashboard designed for caregivers to monitor and manage the older person.

**Methods:**

The HAAL platform comprises a dashboard that integrates, aggregates, and analyzes heterogeneous data gathered from an ecosystem of devices designed for and tested with people with dementia. The study was designed as a technical feasibility pilot to test the HAAL ecosystem in 3 countries: Italy, Taiwan, and the Netherlands, where older people with initial, moderate, and severe dementia were enrolled, respectively. The study was run in 2 stages: the alpha and beta pilot studies aimed to test the second and third prototypes of the platform, respectively.

**Results:**

The alpha test was conducted from March to May 2023, involving 41 end users, of which 13 were people with dementia, 13 were ICs, and 15 were FCs. The beta test was conducted from September 2023 to February 2024, involving 83 end users, of which 26 were people with dementia, 20 were ICs, and 37 were FCs. The results have been elaborated and are supposed to be published by 2026.

**Conclusions:**

The HAAL pilot study was an innovative feasibility study whose primary objectives were to assess reduction in care load for FCs, stress relief for FCs and ICs, and improvement in the perceived quality of life for ICs and people with dementia. The study also evaluated the usability and the acceptance of the platform. Preliminary analyses of the results showed that the HAAL platform partially relieved caregivers’ stress and that the quality of life of people with dementia did not worsen over the test period.

**International Registered Report Identifier (IRRID):**

RR1-10.2196/59860

## Introduction

### Background

Dementia is a chronic neurodegenerative syndrome characterized by deficits in cognitive functions, associated with the loss of daily function and with mental and behavioral disorders. The number of people living with dementia worldwide is about 50 million, and it is thought to become 152 million by 2050 [1]. In Europe, there were 7.7 million cases in 2001 that are expected to double by 2040 [2]. In Taiwan, the trend is even worse: the number of people diagnosed with dementia is expected to double every 20 years, exceeding 0.6 million in 2050 [3]. Neuropsychiatric symptoms such as apathy, social withdrawal, disinhibition, agitation, psychosis, and wandering are a source of stress and negatively affect the quality of life of both the person with dementia and their caregiver [4]. There is no cure for Alzheimer disease, although psychosocial interventions have been shown to be effective in improving the quality of life and psychological well-being of people with dementia [5-8]; moreover, they are considered the treatment of choice for the management of psychological and behavioral disorders [9,10]. Neurocognitive disorders, in particular, dementia, represent a major challenge for society. Efforts to reduce the burden for caregivers, as well as for society at large, are imperative. Nordberg et al [11] showed that the amount of informal care received by people with dementia is as much as 6 times greater than formal care, with supervision constituting the largest proportion of the total informal care provided. Older people generally report higher preferences for their home over other living arrangements, and from the societal point of view, this can contribute to the burden of care, for example, for their families. Dementia caregivers are at high risk of care burden, anxiety, and stress, which exposes them to a higher rate of mortality compared with noncaregivers (Vitaliano et al [12]). Thus, promoting ageing in place for people with dementia should not constitute a strategy to shift the burden of care from the formal care services to the informal caregivers (ICs). Instead, efforts should focus on reducing caregiver stress. Part of the difficulties and stress related to caregiving might be prevented by new information and communication technologies and by developing innovative support services for these people [13,14]. This corresponds closely with the recent European strategy on long-term care [14] as well as the recent Horizon 2020 Work Programme [15], both emphasizing the key role of novel information and communication technology systems in supporting the independent living of older people, as a complement to support from other persons. Previous large-scale evaluations, such as the Whole Demonstrator System in the United Kingdom [16], did not include patients with cognitive impairment, even if, for instance, Alzheimer disease is among the most burdensome diseases for the European Society [17]. These data have the potential to inform regional and national policy makers, allowing the introduction of innovative and cost-effective interventions in order to reduce the burden of Alzheimer disease on public finances and single families.

### The HAAL Project

The Healthy Ageing Ecosystem for People With Dementia (HAAL) project (AAL-2020-7-229-CP, funded by the European Commission) aimed to develop an ecosystem where smart care technologies are embedded in a unique and innovative platform able to support older people ranging from early to late dementia and their caregivers. The services that were developed in HAAL can support people with dementia to stay longer in their own residences with some degree of independence, supporting the final aim of decreasing the workload of caregivers, while improving the quality of care. The project aims to achieve this goal through 4 main actions: care assistance, monitoring, notification, and prevention. Care assistance is provided by supporting the independence of people with dementia on a daily basis and maintaining their well-being. In practice, it is provided through reminders of daily activities, cognitive and physical stimulation, communication with ICs and formal caregivers (FCs), and an alert button for emergency cases. Monitoring is meant for collecting behavioral data continuously to support remote monitoring of people with dementia, in order to provide more targeted care based on data patterns. The information includes lifestyle patterns of activeness, falling status, location, quality of sleep, and performance for rehabilitating exercises of people with dementia.

The notification service sends real-time notifications to caregivers in case of behavioral deviation. The alarm is triggered by data provided through the monitoring device, which could recognize the lifestyle patterns of the users. Behavioral deviation also includes delayed medication and languishes, while emergency cases include falling, wandering, and a patient getting lost. Finally, HAAL invests in prevention to avoid undesirable accidents or irreversible deterioration. This service runs in parallel with the alarm by using longitudinal behavioral data to predict the risk of falling, getting lost, dementia progression, and other frailty symptoms.

The HAAL project stood out from traditional ambient assisted living systems by integrating a customizable, artificial intelligence–driven ecosystem that actively supports both people with dementia and their caregivers. Unlike conventional ambient assisted living solutions, which rely on passive monitoring, HAAL leverages predictive analytics to detect behavioral deviations and cognitive decline patterns over time, allowing for early interventions before critical issues arise. Traditional systems primarily focused on reactive alerts, but HAAL not only offered real-time monitoring but also provided proactive, data-driven insights to help caregivers anticipate risks and adjust care strategies accordingly. Furthermore, while past solutions often followed a one-size-fits-all model, HAAL ensures highly personalized care, dynamically adapting assistive technologies to the specific stage of dementia so that support evolves with the user’s changing needs. In fact, many existing platforms lacked flexibility, whereas HAAL is scalable and modular, integrating different smart devices as required to provide a seamless transition from early to advanced dementia care.

### Goal of the Study

This paper presents the HAAL field trial, a feasibility study whose primary objectives were to assess the stress relief at work for FCs and ICs, the improvement in the perceived quality of life for ICs and persons with dementia, the reduction in care load for the FCs, and the increased cost-effectiveness of the HAAL solution in comparison with the available services. Secondarily, the study aimed to assess the usability and acceptance of the HAAL platform for both people with dementia and caregivers. The purpose of this project was to present and share with the scientific community the HAAL innovative ecosystem and the related field trial protocol for testing it worldwide (Italy, the Netherlands, and Taiwan) with caregivers and people with dementia in real scenarios.

## Methods

### The HAAL Platform

**Figure 1:**
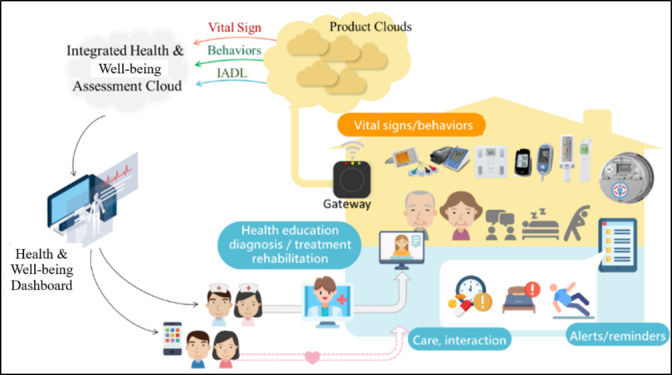
Healthy Ageing Ecosystem for People With Dementia (HAAL) platform. IADL: instrumental activities of daily living.

The HAAL platform, presented in this section, is comprises 6 smart technologies designed for people affected by dementia (an interactive game for cognitive training, a social robot, a GPS-tracking system, a lifestyle-monitoring system, an alarm system, and a smart mattress) and 1 dashboard—consisting of a web app—to be installed on a tablet or PC. Since people with dementia start losing the ability to comprehend some digital interactions (social robots and interactive games) in the transition from early to middle dementia, each user benefits from a combination of such devices, according to their own needs. The dashboard, which is used by the IC, receives, analyzes, and shows data acquired and processed by those devices through machine learning (ML) algorithms to detect and determine the health status of people with dementia. In particular, unsupervised algorithms (k-means) were adopted to highlight the deviation of people with dementia from standard behavior, while supervised algorithms were applied to describe and predict the behavior of people with dementia. The first prototype of the platform has been preliminarily tested in the laboratory by Morresi et al [18] and then underwent the usability test successfully [19]. Then, subsequent prototypes 2 and 3 were developed to be tested in the alpha and beta stages of experimentation, respectively. The alpha prototype was developed on the basis of integrated fictitious data, used for formative evaluation with the goal of studying the overall experience, usability, acceptability, and attitude toward the system over a short time period to collect feedback from end users. The beta prototype was a version of the system intended for market launch that includes ML algorithms to predict various aspects of the well-being of people with dementia such as the previous version but refined through data collected during the alpha testing stage of the project. In summary, while the alpha prototype was trained with simulated data, the beta prototype implemented ML algorithms with real data. In addition to that, much of the feedback received at the end of the alpha stage was translated into technical requirements and implemented in the subsequent beta version to improve the usability, acceptability, and efficiency of the platform. Figure 1 shows the platform architecture.

#### WhizToys

**Figure 2:**
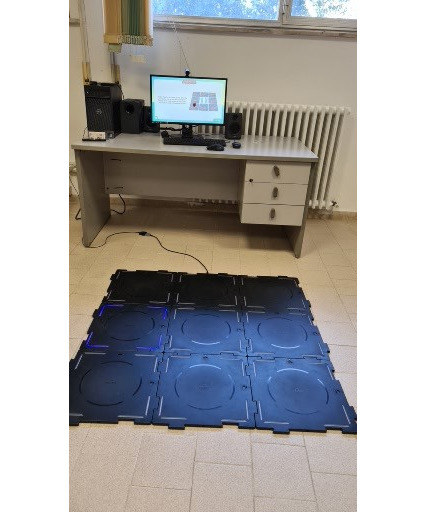
WhizToys.

WhizToys, displayed in Figure 2, is 9 lightweight, portable motion-sensing floor tiles arranged to form a 3 × 3 square. They can be connected to a television to display game instructions on the screen. The game combines physical activity and cognitive training. The game is considered a serious gaming gym that can be personalized. This could prevent cognitive function decline or slow down the progression of the mild cognitive impairment stage. Older adults could participate in different cognitive games, such as music, numbers, colors, and spelling, simply by walking and stepping on tiles. In addition to providing multisensory stimulation such as visual, light, and sound, the app also allows caregivers to choose a game type and difficulty level. The app also provides individual user accounts and stores the game results in the cloud for subsequent evaluation and analysis. Basically, the input variables include people’s movement and their background information such as age and gender. The primary output of the WhizToys game app includes steps, total playtime for a game, memory time for the memory-training games, frequency of use, correction rate of the game, length of steps, and time of the timed up and go results. The app also provides individual user accounts and stores the game results in the cloud for subsequent evaluation and analysis.

#### Tinybots

**Figure 3:**
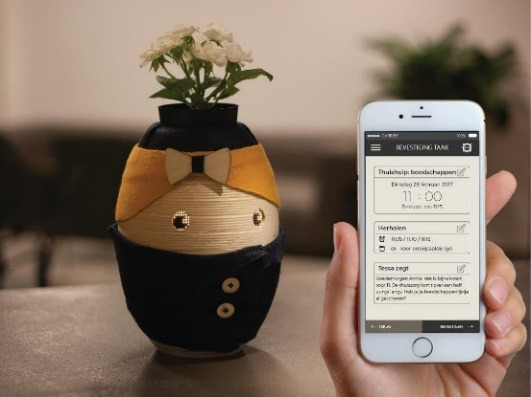
Tinybots and its web application.

Tinybots, displayed in Figure 3, is a small social robot named Tessa, able to provide verbal guidance to older adults on daily activities. Tessa has her own speaker and microphone to communicate with the user and is able to understand yes/no answers. Tessa helps people with orientation by giving personal reminders, suggesting activities, or providing instructions for certain tasks. Tessa helps people get active again by providing spoken suggestions and by playing personal music. In fact, caregivers can schedule the tasks and personalize the spoken messages and instructions spoken by Tessa, which helps people with dementia to structure their days. ICs use a simple web app to write messages for Tessa and schedule these messages at specific times.

#### Kompy Pico

**Figure 4:**
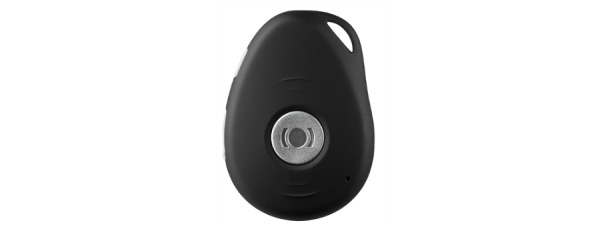
Kompy Pico.

Kompy Pico, displayed in Figure 4, is a GPS tracker that can be used to localize or call someone and to set a geofence. It is a personal device that the user can wear as a necklace, attach to a belt, or keep in a pocket, and it has a GPS, Bluetooth, and Wi-Fi. The device is precise in determining the location both in indoor and outdoor locations. Kompy Pico provides direct communication between the people with dementia and the IC, which can be called through the alarm button. The caregiver receives the alarm notification directly on the mobile app Mopas, available on both Google Play and Apple Store. As soon as the alarm goes off, the exact position of the people with dementia is sent to the caregiver. The caregiver can set a “safe zone,” which is a specific area outside of which the user is not expected to be and therefore the alarm can be sent. All the activities of the people with dementia for the last 60 days can be tracked and logged in the app.

#### Sensara

**Figure 5:**
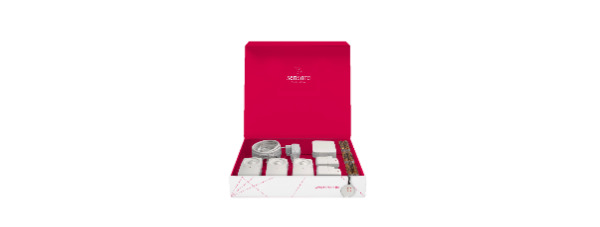
Sensara.

Sensara HomeCare has 8 wireless sensors that can be installed in toilets or bathrooms, kitchens, exit doors, halls, and living rooms to monitor older persons’ daily activities (Figure 5). The sensors are unobtrusive, battery operated, and privacy friendly. Due to smart (self-learning) algorithm design, the system automatically supports all possible customer lifestyles. After 2 weeks of use, the algorithm is able to distinguish the person’s daily activities and set a baseline behavior. Sensara informs the FCs and ICs via a smartphone app if something goes wrong. When caregivers want to zoom in on trends, they can see how things are going in the long run. Otherwise, they can check on what is going on in the present on the events list.

#### CogvisAI

**Figure 6:**
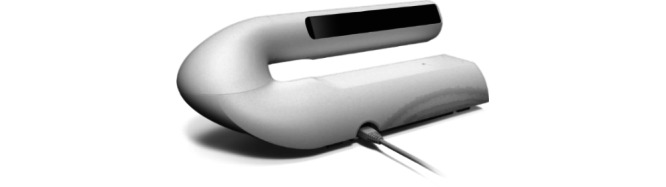
CogvisAI.

CogvisAI, displayed in Figure 6, is a medium-sized sensor to be mounted on a wall of a room that makes use of 3D smart sensors to analyze behavior. Based on these data, an alarm can be sent in critical situations: when an inhabitant leaves the space or falls. Furthermore, it can be sensed if someone is in the bed or not. CogvisAI mainly focuses on the later stage of dementia, in which older adults have a problem wandering or falling. Furthermore, the device is coupled to a specific account from which the IC can see the status of the device and settings for which detections a notification should be sent to whom.

#### WhizPad

**Figure 7:**
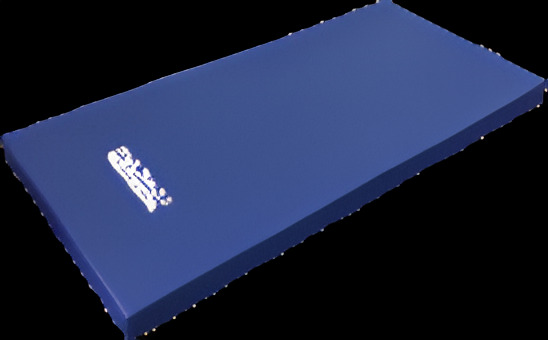
WhizPad.

WhizPad is a comfortable mattress made with temperature-sensitive pressure-relieving foam to prevent pressure ulcers (Figure 7). WhizPad is a thin mattress pad made of memory foam and conductive textile materials. WhizPad counts more than 30 sensing areas installed, which provide an on/off signal, which means that whenever they are activated, they provide a binary output (on = sensor is activated, off = sensor is not activated). Data coming from pressure sensors are processed and fed into an ML algorithm for posture identification of the patient. Given the event algorithms implemented in a bedside data processor, the pressure signals collected by WhizPad can be used to detect on/off bed, sleep posture, movement counts, and respiration rate. Integrated with information and communication systems, caregivers can maintain awareness of older adults’ daily activities and needs by using their mobile devices to access the WhizPad for real-time monitoring and a historical data record of bed-related activities, as well as receiving service reminders and alerts for abnormal events.

#### Dashboard

**Figure 8:**
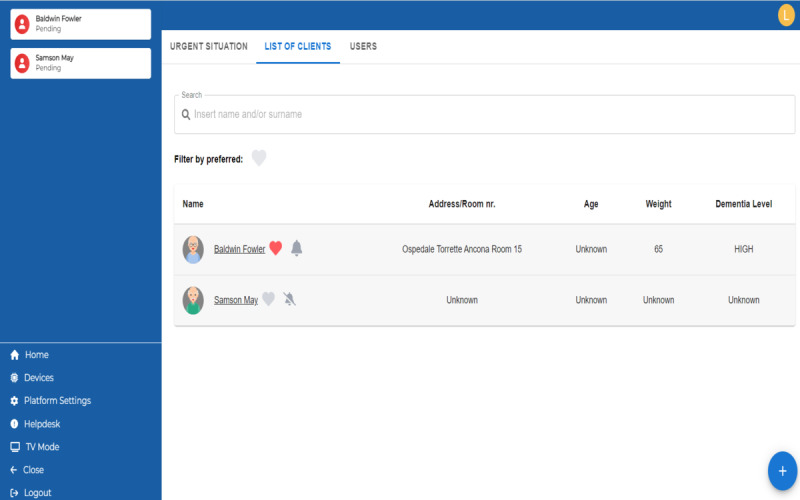
Healthy Ageing Ecosystem for People With Dementia (HAAL) dashboard home page: lists of patients followed by a caregiver.

**Figure 9:**
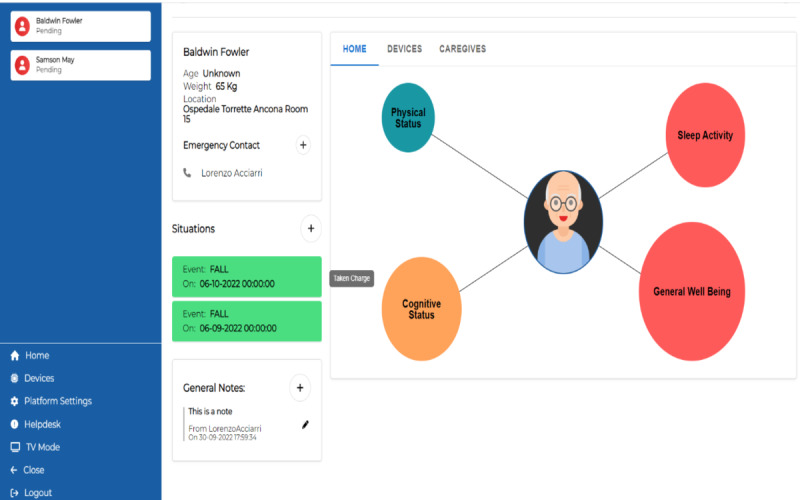
Healthy Ageing Ecosystem for People With Dementia (HAAL) dashboard. General overview of a single patient’s status: quality of physical status, sleep activity, cognitive status, and general well-being.

**Figure 10:**
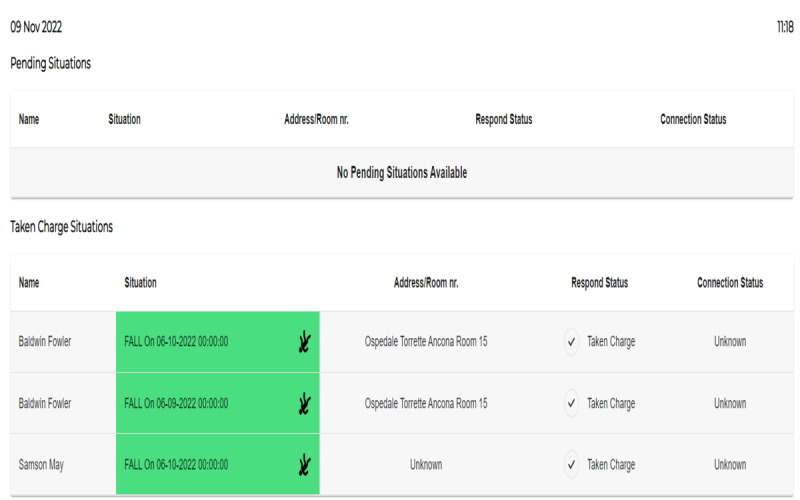
Healthy Ageing Ecosystem for People With Dementia (HAAL) dashboard. Overview of situations occurred to a patient.

**Figure 11:**
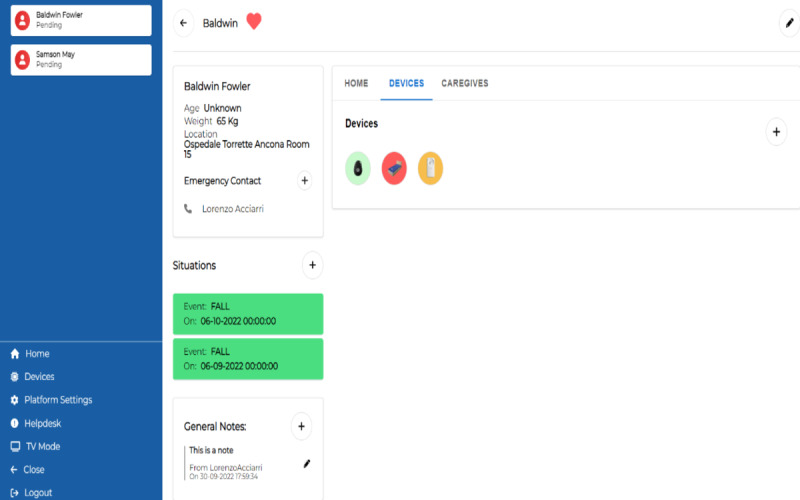
Healthy Ageing Ecosystem for People With Dementia (HAAL) dashboard. Overview of installed devices used of a patient.

The core of the HAAL project is the development of the dashboard, which focuses on the well-being of people with dementia. In fact, the dashboard integrates heterogeneous data from the abovementioned smart care products and is processed by an ML algorithm. The dashboard primarily focuses on descriptive analyses with relatively low complexity and a low level of automation. The indices provided by the smart care products are categorized into “vital sign indices,” “behavior indices,” and “instrumental activities daily living indices.” Based on public standards (ie, set by World Health Organization) and personal patterns (established by everyday measurements), the assessment algorithm ranks the indices into 4 levels: great, normal, attention, and abnormal. The final status is marked as the worst level among all 4 statuses. Notably, each profile has 4 statuses: high urgency (need action at that time), low urgency (may need attention), offline (adopted devices are not working or connected), and normal (no detected deviation). These statuses are shown in 4 colors: red, orange, blue, and green, respectively. Besides, the deviations or accidents require action from FCs. Each situation contains 3 levels of response status: requiring (need responding), attending (when a caregiver responds), and being already attended (when a caregiver marks the situation as safe). The second branch for medical workers would focus on giving a quick overview of several clients and urgent notifications. The dashboard is designed to be displayed on a television or a personal working computer. The notification is sent to a working mobile phone. The health and response statuses are consistent with the first branch. Figures 7-11 show various screens of the dashboard to understand what the caregiver can see and do on it. The caregiver, through the menu “List of Clients” (shown in Figure 8), has an overview of clients whom he or she is caring for. Moreover, there are data about age, weight, level of dementia, and location of the patient.

Then, the caregiver can select 1 single patient and get an overview of its situation, coded by color and dimension of the following aspects: physical status, sleep activity, cognitive status, and general well-being, as shown in Figure 9. In the same screen (Figure 9), the caregiver has information about pending situations, general notes (that are added by the caregiver), and emergency contacts of the patient.

In the “Situations” window, shown in Figure 10, the caregiver can view who requires intervention. In this case, all the situations were already taken in charge (in fact, the situations are shown in green). The kind of situation is written in the column “situation,” and the location and connection status are shown.

In the device menu, displayed in Figure 11, it is possible to see which devices are used by the patient. The color (given by the round of the device) codes the level of alert related to each device. In Figure 11, the analysis of data collected by WhizPad is alarming (red), whereas there is a warning related to Sensara, and no problem from the GPS tracker.

**Figure 12:**
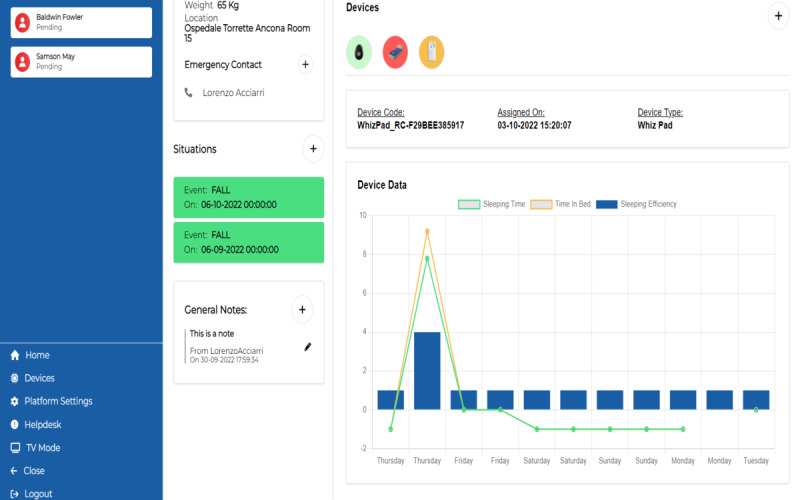
Healthy Ageing Ecosystem for People With Dementia (HAAL) dashboard. Focus on data analysis of a device: measurement of sleep quality by WhizPad.

Then, the caregiver can explore a single device to get insights about the collected and analyzed data. In Figure 12, an analysis of data collected by WhizPad is shown.

### The Field Trial

The field trial was conducted as an uncontrolled longitudinal study, with a before and after design where the observations are made on a series of enrolled individuals, receiving the intervention described in this section with no control group, with data collected before and after the installation and use of the technical solution. The uncontrolled longitudinal design was chosen to assess the practical implementation, usability, and preliminary effectiveness of the HAAL platform, although it might introduce selection bias, measurement bias, and confounding variables. The field trial was also conducted in 2 stages—alpha and beta—where prototypes 2 and 3 of the HAAL dashboard were tested, respectively. Both phases were run in the same way in terms of duration and activities carried out, as well as interviews. The field trial procedure was divided into 4 different phases after the recruitment of the participants: baseline evaluation (T1), first evaluation (T2), second evaluation (T3), and final evaluation (T4), with the aim of collecting data as described in Textbox 1. Given the study’s reliance on consistent participation from people with dementia and their caregivers, data dropouts were anticipated and addressed by design. In fact, as the study involves 4 evaluation phases, partial data could still be collected and analyzed even if a participant dropped out. The same principle stands for qualitative data. However, to prevent dropout and make the participants feel involved in the study, they were contacted frequently (eg, every 2 weeks) by research personnel and supported promptly any time they needed technical support. In any case, even dropouts, accompanied by justification, constitute useful feedback in a feasibility study in order to understand the weaknesses of the solution to be improved.

**Field trial phases.**

- Recruitment phase: the recruitment protocol included general information on the subjects, in particular, health status and cognitive condition. The information was collected with the help of the caregiver or family member, if needed.
- Baseline evaluation consisted of the first real contact with the users and their families, before the start of the field trial.
- First evaluation (after 1 month of use): the aim of this phase is to analyze the usability and acceptability of the system, after a short period of use. The evaluation of the system usability was conducted adopting qualitative and quantitative techniques.
- Second evaluation (after 2 months of use): the aim of this phase is to analyze the usability and acceptability of the system, after a short period of use. The evaluation of the system usability was conducted adopting qualitative and quantitative techniques.
- Final evaluation (after 3 months of use): the aim of this phase is to collect useful information on the whole benefits perceived by the users after a meaningful period of use of the system. The final evaluation was conducted after the system deinstallation to detect and analyze the impact of the system in the daily life of the older people and their family and to gain knowledge on older adults’ technology acceptance and usability issues and provide methodological approach for further studies in the field.

### Recruitment

In the dementia care context for HAAL, 3 groups of end users were involved, such as people with dementia, ICs, and FCs. People with dementia vary between mild cognitive impairment and severe stages of dementia. This intellectual deterioration can be measured using the Global Deterioration Scale, which defines 7 cognitive decline stages and 4 stages of dementia [20]. As this was a pilot study on the feasibility of using the technology, the sample size was not calculated. However, similar studies [21,22] were taken as reference points and, above all, the analysis of Billingham et al [23], which shows that in feasibility pilot studies, net of great variability, an average of 30-36 participants are included. For these reasons, it was decided to recruit 45 participants in the alpha test and 90 participants in the beta test, that is, 15 and 30 triads, respectively, equally divided among the 3 pilot sites.

In Italy, the end users were recruited from the neurology unit and Alzheimer assessment unit (Memory Clinic) of the Italian National Institute of Health and Science on Aging (IRCCS INRCA). The research team had the opportunity to meet the families of patients with dementia and to present them with the project and the modalities of possible participation. Researchers at Vilans—a private organization for digital health—recruited participants via the care organization Livio, established in the Enschede region, in the Netherlands. In Taiwan, 2 research institutes actively participated in the HAAL field trials. The Yuan Ze University recruited participants at Bianciao Veteran Dementia Nursing Home, while the National Cheng Kung University recruited the end users from day care centers established by Schuhe Social Welfare Foundation.

#### Older People With Dementia

Once the informed consent was obtained in duplicate, the compliance with the criteria of inclusion and exclusion of the study was verified, and the baseline evaluation was carried out with the questionnaires and clinical trials provided by the study design. The inclusion and exclusion criteria are reported in Textbox 2.

**Inclusion and exclusion criteria for people with dementia.**

**Inclusion criteria**

- Aged 65 years and older.
- Capacity to consent.
- Score at the Global Deterioration Scale (GDS) as follows: GDS 2-4 (group A), GDS 5 (group B), and GDS 6-7 (group C).
- Have both an informal and a formal caregiver to support in carrying out the main daily activities.
- Healthy sight and hearing.

**Exclusion criteria**

- Active implantable medical device incorporated.
- Failure to meet the inclusion criteria.
- Concomitant participation in other studies.
- Lack of written informed consent.
- Lack of informal caregiver.
- Sight and hearing not intact.

#### Informal Caregiver

ICs are mostly family members or daily references of the people with dementia. Connections between ICs and clients are private and not via a care organization. ICs are usually concerned about the well-being of the people with dementia, and they provide emotional or practical support regularly. Although it is easier to live near the people with dementia, ICs did not necessarily need to live close to the people with dementia. ICs were included in the HAAL project since they play an important role in the daily life of the people with dementia. Moreover, the ICs can fulfill caring tasks to reduce the burden of FCs. A good collaboration between IC and FC is needed, as the IC can be the link between the client and the FC.

The inclusion and exclusion criteria are reported in Textbox 3.

**Inclusion and exclusion criteria for informal caregivers.**

**Inclusion criteria**

- Being the informal caregiver of a person with cognitive impairment or dementia in stage, following the Global Deterioration Scale groups.
- Availability of time to participate.
- Visiting the assisted person at least 2 times a week or living with him or her.

**Exclusion criteria**

- Active implantable medical device incorporated.
- Lack of familiarity with apps and minimal digital literacy.
- Failure to meet the inclusion criteria.
- Concomitant participation in other studies.
- Lack of written informed consent.

#### Formal Caregivers

Care professionals or FCs are professionally responsible for dementia care. They are trusted agents for people with dementia and have direct contact with older adults and provide care when needed. In Italy, the FCs are a case manager, neurologist, psychotherapist, occupational therapist, and nurse. The case manager is responsible for the organization. The neurologist is a specialized medical doctor who diagnoses the disease and prescribes the treatment. The psychotherapist leads the intervention, whereas the nurses and the occupational therapists act as assistants. These professionals may be especially found in day care, but they are available in residential home care as well. People at a severe stage of dementia are suggested to move to a care home.

In the Netherlands, the FCs are case managers or nurses. In other words, they are local district nurses, and case managers have a specialization in dementia care. Nurses are responsible for nondiagnosed seniors, while case managers would be assigned for the diagnosis of people with dementia and take part in long-term care provision. They will provide home care in the early and middle stages of dementia until the person needs more intensive professional care for daily tasks. Then, in the severe stage, the case managers would suggest the transition to a care home to receive professional caregiving.

In Taiwan, the care of people with dementia is first evaluated by the case managers of the long-term care management centers supervised by Ministry of Health and Welfare. The case managers are well-trained physicians, physical therapists, occupational therapists, nurses, and social workers. After the assessment, the case managers will arrange related follow-up care services for people with dementia. The follow-up care services include home care, day care centers, institutional care, group homes, psychiatric wards for older adults, institutional respite services, and so forth (Textbox 4).

**Inclusion and exclusion criteria for formal caregiver.**

**Inclusion criteria**

- Being the formal caregiver of a person with cognitive impairment or dementia in stage, following the Global Deterioration Scale groups.
- Availability of time to participate.
- Work experience of at least 4 years in the field.

**Exclusion criteria**

- Failure to meet the inclusion criteria.
- Concomitant participation in other studies.
- Lack of written informed consent.

### Outcomes

The field trials aimed to assess the feasibility of implementing the HAAL platform in the assistance of people with dementia at any stage. For this reason, the primary interest was to assess the impact of the HAAL platform on caregivers’ workload and end users’ quality of life, as described in Textbox 5. Then, the field trials also focused on the usability and cost-effectiveness of the HAAL solution, as shown in Textbox 6.

**Primary outcomes of the study.**

- Reduction of the care load of the formal caregiver (FC) and the informal caregiver (IC) through Zarit Burden Interview [24].
- FC’s and IC’s decrease in stress and anxiety through the General Anxiety Disorder-7 [25].
- Improvement in the perceived quality of life for IC and person with dementia through EQ-5D [26].

**Secondary outcomes of the study.**

- Usability of the platform for formal caregiver and informal caregiver through System Usability Scale [27] and semistructured interview.
- Increased cost-effectiveness of the Healthy Ageing Ecosystem for People With Dementia (HAAL) solution in comparison with the available services through semistructured interview.

For these reasons, the protocol included study-specific questions on demographics, attitudes toward and acceptance of the HAAL platform technology, and questions regarding demands and cost. All scales used are validated in the pilot sites’ languages and suitable for administration to the patients recruited in the study. Tables 1-Tables 4 summarize different tools adopted with each end user group in all the phases of the study.

**Table 1:** Tools and dimensions for the people with dementia protocol at stages 2-4 of the Global Deterioration Scale.

Dimension and tool			R^a^		T1^b^		T2^c^		T3^d^		T4^e^
Cognitive status: GDS^f^ [20]			✓								✓
Sociodemographics: ad hoc questions					✓						
eHealth literacy: eHEALS^g^ [28]					✓						
Attitude toward technology: ATDPA-E^h^ [29]					✓						
Quality of life: EQ-5D [26]					✓		VAS^i^		VAS		✓
**Usability**											
	SUS^j^			✓		✓		✓		✓	
	Ad hoc questions					✓				✓	
	Desirability cards					✓				✓	
Acceptability: ad hoc questions											✓

^a^R: recruitment.

^b^T1: baseline.

^c^T2: first evaluation.

^d^T3: second evaluation.

^e^T4: final evaluation.

^f^GDS: Geriatric Depression Scale.

^g^eHEALS: eHealth Literacy Scale.

^h^ATDPA-E: Assistive Technology Device Predisposition Assessment, Scale E.

^i^VAS: Visual Analogue Scale.

^j^SUS: System Usability Scale.

**Table 2:** Tools and dimensions for the people with dementia protocol at stages 5 and 6-7 of the Global Deterioration Scale.

Dimension	Tool	R^a^	T1^b^	T2^c^	T3^d^	T4^e^
Cognitive status	GDS^f^ [20]	✓				✓
Sociodemographics	Ad hoc questions		✓			
Quality of life	EQ-5D [26]		VAS^g^	VAS	VAS	VAS
Usability	Ad hoc questions			✓		✓
Acceptability	Ad hoc questions					✓

^a^R: recruitment.

^b^T1: baseline.

^c^T2: first evaluation.

^d^T3: second evaluation.

^e^T4: final evaluation.

^f^GDS: Geriatric Depression Scale.

^g^VAS: Visual Analogue Scale.

**Table 3:** Tools and dimensions of the informal caregiver protocol.

Dimension and tool		R^a^	T1^b^	T2^c^	T3^d^	T4^e^
Sociodemographics: ad hoc questions		✓	✓			
eHealth literacy: eHEALS^f^ [28]			✓			
Attitude toward technology: ATDPA-E^g^ [29]			✓			✓
Social support: Lubben Scale [30]			✓			
Quality of life: EQ-5D [26]			✓	VAS^h^	VAS	✓
Caregiver burden: Zarit Burden Interview [24]			✓	✓	✓	✓
Anxiety and stress: GAD-7^i^ [25]			✓	✓	✓	✓
**Usability**						
	SUS^j^ [27]			✓		✓
	Ad hoc questions			✓		✓
	Desirability cards			✓		✓
Acceptability: ad hoc questions						✓
Demand and cost information: ad hoc questions						✓

^a^R: recruitment.

^b^T1: baseline.

^c^T2: first evaluation.

^d^T3: second evaluation.

^e^T4: final evaluation.

^f^eHEALS: eHealth Literacy Scale.

^g^ATDPA-E: Assistive Technology Device Predisposition Assessment, Scale E.

^h^VAS: Visual Analogue Scale.

^i^GAD-7: 7-item Generalized Anxiety Disorder.

^j^SUS: System Usability Scale.

**Table 4:** Tools and dimensions of the formal caregiver protocol.

Dimension and tool		R^a^	T1^b^	T2^c^	T3^d^	T4^e^
Sociodemographics: ad hoc questions		✓	✓			
eHealth literacy: eHEALS^f^ [28]			✓			
Attitude toward technology: ATDPA-E^g^ [29]			✓			✓
Quality of life: EQ-5D [26]			✓	VAS^h^	VAS	✓
Caregiver burden: Zarit Burden Interview [24]			✓	✓	✓	✓
Anxiety and stress: GAD-7^i^ [25]			✓	✓	✓	✓
**Usability**						
	SUS^j^ [27]			✓	✓	✓
	Ad hoc questions			✓		✓
	Desirability cards			✓		✓
Acceptability: ad hoc questions						✓
Demand and cost information: ad hoc questions						✓

^a^R: recruitment.

^b^T1: baseline.

^c^T2: first evaluation.

^d^T3: second evaluation.

^e^T4: final evaluation.

^f^eHEALS: eHealth Literacy Scale.

^g^ATDPA-E: Assistive Technology Device Predisposition Assessment, Scale E.

^h^VAS: Visual Analogue Scale.

^i^GAD-7: 7-item Generalized Anxiety Disorder.

^j^SUS: System Usability Scale.

### Statistical Analysis

The first step of the data analysis dealt with the description of the sample. Continuous variables were reported as either mean and SD or median and IQR on the basis of their distribution (assessed using Kolmogorov-Smirnov test). Categorical variables were expressed as an absolute number and percentage. Mann-Whitney *U* tests (for nonnormal distribution), or chi-square tests (normal or nonnormal), were used to compare the independent and dependent variables between the preconditions and postconditions, in addition to simple descriptive statistics (means, medians, and SDs as appropriate).

To verify the achievement of the primary end point (ie, caregivers’ burden), subscales of the Zarit Burden Interview questionnaire were calculated. Means and SD or medians and IQRs of the scores were reported according to their distribution. Correlation coefficients (Pearson for normally distributed variables and Spearman for nonnormally distributed variables) of the subscales with the other rating scales at each stage of the study and with the main characteristics of the subjects were calculated to check for potential determinants of higher acceptability, as a secondary end point of the project.

### Ethical Considerations

The study was approved in all the 3 pilot countries: by the ethics committee of the IRCCS INRCA in Italy (3750/2023), in the Netherlands (NW2023-13), and by the institutional review board of the National Cheng Kung University Hospital in Taiwan (B-ER-112-026). The study protocol was registered on ClinicalTrials.gov (NCT06307197).

The principles of the Declaration of Helsinki [31] and Good Clinical Practice guidelines were adhered to. All participants in this study provided written informed consent, but the acquisition of informed consent from people with dementia is a long-debated issue. Although dementia leads to cognitive decline, a diagnosis alone does not imply loss of decision-making capacity. Legal capacity remains unless formally revoked, and people with dementia may retain decision-making skills for a long time. While executive functions deteriorate progressively, early-stage patients may still comprehend simplified consent forms and express preferences aligned with their values. Informed consent requires full understanding and voluntary decision-making. Every effort was made to respect residual autonomy, adapting information to cognitive abilities and facilitating communication. The will of people with dementia took precedence over family input, and consent was continuously monitored throughout the study. Participants could withdraw at any time without consequences. If a participant could not provide valid consent, a legally designated representative will be involved, but the individual’s willingness to participate will still be considered under a “double consent” approach. The study ensured informed decision-making while prioritizing the well-being of participants.

Personal data collected during the trial were handled and stored in accordance with the General Data Protection Regulation, the European law on privacy. For this purpose, data were anonymized at the end of the study. Use of the study data was and will be controlled by the principal investigator (RB). All data and documentation related to the trial are stored in accordance with applicable regulatory requirements, and access to the data is restricted to authorized study personnel.

The equipment needed electric power. However, all devices were of low power consumption. Some of them (tablet, mobile router, and Kompy Pico) are rechargeable. The user was informed on how much electric power is supposed to be consumed in relation to the set of devices. No refund to the end user was given for participating or power consumption.

## Results

The recruitment started in November 2022 and continued until April 2023. The alpha test was conducted from March 2023 to May 2023 involving 41 end users, of which 13 were people with dementia, 13 were ICs, and 15 were FCs. The beta test was conducted from September 2023 to February 2024 involving 83 end users, of which 26 were people with dementia, 20 were ICs, and 37 were FCs. The recruited end users are reported in Table 4 grouped by their category, phase of study, and country.

The efficacy of the HAAL ecosystem will be demonstrated in a separate publication expected to be published by 2026. The HAAL ecosystem, successfully updated from prototype 2 to prototype 3, showed promising results. The use of the system did not negatively impact the caregivers’ burden; in some cases, the stress lowered instead. This positive outcome was coherent with the usability, which received positive scores from the end users. As regards the quality of life of people with dementia, the level remained stable through the period of testing, given that the pathological condition was considered a very positive result. Table 5 shows the end users recruited in all pilot countries in both test stages.

**Table 5:** End users recruited for the field trials in the 3 pilot sites.

	Italy, n		The Netherlands, n			Taiwan, n	
	Alpha	Beta	Alpha	Beta	Alpha		Beta
People with dementia, n	3	7	5	9	5		10
GDS^a^ 2-4	None	None	None	None	5		10
GDS 5	3	7	None	None	None		None
GDS 6-7	None	None	5	9	None		None
IC^b^	3	7	5	3	5		10
FC^c^	5	9	5	18	5		10
Total	13	23	15	30	15		30

^a^GDS: Global Deterioration Scale.

^b^IC: informal caregiver.

^c^FC: formal caregiver.

## Discussion

The preliminary findings from the HAAL feasibility study suggest promising trends regarding the usability, acceptance, and potential benefits of the platform for both people with dementia and their caregivers. The system was tested across multiple pilot sites, providing insights into caregiver burden, stress management, and user interaction with assistive technologies. Results indicate that the HAAL ecosystem was generally well received, with variations in usability perceptions across different user groups and settings. Caregivers reported engagement with the platform and its components, while qualitative feedback highlighted areas for improvement in usability and system integration. Additionally, the study provided valuable learnings on user adaptation to the technology over time. The study design allowed for testing the solution for a relatively long period (3 months) and to exploit feedback coming from the alpha phase to improve the prototype that is then tested in the beta phase. The same process has been done previously through a usability test in which professional workers tested the dashboard prototype 1 [19]. Thus, this protocol applied to prototypes 2 and 3 of the HAAL dashboard. The iterative user-centered design has characterized all the HAAL project stages, starting from cocreation with end users (both primary, ie, FC, and secondary, ie, people with dementia) till the presented tests. The main challenges of this study were represented by the cultural and health care systems differences among the 3 pilot sites and 2 continents. The HAAL platform was ambitious as it aimed to work for managing people of any degree of dementia in different care settings. For these reasons, and given the low number of end users recruited, it has been decided to recruit only 1 category of people with dementia in each pilot site. In fact, this methodology allowed for the reduction of the number of variables to consider in data analysis.

The HAAL platform is designed to be scalable through the use of Amazon Web Services, which allows it to manage a large number of devices and users. Integrating different devices and heterogeneous systems is a key challenge of the HAAL project, which took a modular and flexible approach, offering application programming interfaces to enable partners to natively integrate with the ecosystem. Regarding off-the-shelf products, specific software integration microservices were developed. This heterogeneity requires device-specific normalization of information, linking each device to a specific client at a specific time interval. In addition, the lack of event standardization and the difficulty in monitoring the proper connection of devices are further limitations to interoperability. Although the HAAL platform was designed with scalability and interoperability features, its practical implementation presented challenges related to device heterogeneity and the need for customization. Future efforts should focus on standardization, reliable data collection, and integration of user feedback to overcome these limitations. In fact, several technological devices are available on the market and tested for this target group; however, very few ecosystems are in place and used by health care facilities, institutions, or systems. In this regard, the challenge was to embed and aggregate different technologies to provide quick and intuitive information that can effectively support caregivers in managing people with dementia, relieving their stress partially. Moreover, the need extends beyond mere access to information; it encompasses the development of comprehensive support systems that cater to the dynamic needs of caregivers and people with dementia alike. These systems must go beyond the provision of basic information and extend to offering personalized guidance, respite services, and community resources. Using the user-centered design approach, with input from caregivers throughout the development process, the design team worked to make the dashboard as usable as possible. End users received a brief lesson on how to use the dashboard along with a handbook. The aim of this approach was to avoid frustration and additional burden to the care of people with dementia. Moreover, the measurement of technological skills (Assistive Technology Device Predisposition Assessment) and digital health literacy (eHealth Literacy Scale), along with usability (System Usability Scale), acceptability (Unified Theory of Acceptance and Use of Technology), and burden (Zarit Burden Interview), allows us to assess whether additional burden due to learning and using a new dashboard depended on the caregiver’s digital and health literacy. By fostering a collaborative environment where caregivers feel empowered and supported, these initiatives not only enhance the quality of care provided but also contribute to the overall well-being of both caregivers and care recipients. Within this framework, the main objective of the HAAL project is to assess the stress relief at work for FCs and ICs and the improvement in the perceived quality of life for ICs and persons with dementia.

## Abbreviations

FC: formal caregiver
HAAL: Healthy Ageing Ecosystem for People With Dementia
IC: informal caregiver
IRCCS INRCA: Italian National Institute of Health and Science on Aging
ML: machine learning

## Data Availability

Data are not available to share due to Department of Veterans Affairs and ethical retractions; however, annotation instructions and vocabulary are available upon request..
